# Prognostic value of the prognostic nutritional index for patients with acute myocardial infarction undergoing percutaneous coronary intervention with variable glucose metabolism statuses: a retrospective cohort study

**DOI:** 10.1186/s13098-023-01160-4

**Published:** 2023-10-24

**Authors:** Xuebin Ling, Chufen Lin, Jin Liu, Yibo He, Yongquan Yang, Na Lu, Wei Jie, Yong Liu, Shiqun Chen, Junli Guo

**Affiliations:** 1 Hainan Provincial Key Laboratory for Tropical Cardiovascular Diseases Research, Department of Cardiovascular Medicine of The First Affiliated Hospital, Key Laboratory of Emergency and Trauma of Ministry of Education, Hainan Medical University, Haikou, China; 2 Department of Health Medicine, The Affiliated Haikou Hospital of Xiangya Medical College, Central South University, Haikou, China; 3 Department of Cardiology, Guangdong Cardiovascular Institute, Guangdong Provincial People’s Hospital, Guangdong Academy of Medical Sciences, Guangzhou, China

**Keywords:** Prognostic value, Prognostic nutritional index, Acute myocardial infarction, Percutaneous coronary intervention, Glucose metabolism statuses

## Abstract

**Background:**

The prognostic nutritional index (PNI) and different glucose metabolisms have been separately reported to be correlated with long-term prognosis in patients with acute myocardial infarction (AMI) undergoing percutaneous coronary intervention (PCI). However, PNI application in patients with an impaired glucose metabolism has not been well validated, especially in pre-diabetic patients. This study evaluated whether PNI influences a long-term risk of mortality along different glucose metabolism statuses.

**Methods:**

A total of 17,697 patients with AMI and a history of PCI were enrolled in this retrospective observational cohort study from January 2007 to December 2020. Three subgroups with different glucose metabolism statuses, including normal glucose regulation (NGR), pre-diabetes mellitus (pre-DM), and diabetes mellitus (DM), were divided into three groups according to the tertiles of PNI, respectively.

**Results:**

All-cause mortality occurred in 2613 (14.8%) patients within a median of 4.1 years of follow-up. Upon analyzing the Kaplan–Meier plots for the NGR, pre-DM, and DM groups, the incidence of all-cause or cardiovascular mortality in the low PNI (PNI-L, ≤ 42.7) subgroup was significantly higher than that in the median PNI (PNI-M, > 42.7 and ≤ 48.2) and high PNI (PNI-H, > 48.2) subgroups (all, *P* < 0.001). After adjusting for confounding factors, the hazard ratio (HR) for all-cause mortality in the PNI-L group significantly increased compared to that in the PNI-H subgroups of the NGR group (HR, 1.35; 95% CI 1.14–1.66; *P* < 0.001), pre-DM group (HR, 1.29; 95% CI 1.02–1.62; *P* < 0.001), and DM group (HR, 1.36; 95% CI 1.13–1.63; *P* < 0.001). Given that there was evidence of interactions between PNI and different glucose statuses (*P* for interaction < 0.001), patients were divided into nine subgroups, and we found that DM patients with PNI-L statuses had the highest risk of all-cause mortality compared to NGR patients with PNI-H statuses (HR, 1.69; 95% CI 1.42–2.01; *P* < 0.001).

**Conclusion:**

Lower PNI is a significant and independent risk factor for all-cause mortality in AMI patients undergoing PCI with different glucose metabolism statuses, and this risk further increases with DM compared to NGR or pre-DM statuses.

**Supplementary Information:**

The online version contains supplementary material available at 10.1186/s13098-023-01160-4.

## Introduction

Early revascularization in patients with acute myocardial infarction (AMI) can save the dying myocardium, but reperfusion myocardium can cause myocardial ischemia–reperfusion injury (MIRI) due to endothelial cell dysfunction, inflammatory reactions, oxygen free radical injury, and calcium overload [1]. MIRI is associated with more than 50% of postoperative adverse cardiovascular events, which seriously affects the quality of life and the prognosis of patients [2]. The disorder of glucose metabolism can further aggravate MIRI. Studies have suggested that admission hyperglycemia measurements in patients with AMI undergoing percutaneous coronary intervention (PCI) can cause inflammatory reactions and oxidative stress injury, aggravate endothelial dysfunction, block coronary artery blood flow, and increase the infarct size, which are significantly related to the long-term adverse event outcome [3]. Using our Cardiorenal ImprovemeNt-II (CIN-II) study (ClinicalTrials.gov identifier NCT05050877) database, a total of 23,162 patients with AMI undergoing PCI were divided into normal glucose regulation (NGR) (n = 10,456), pre-diabetes (pre-DM) (n = 4929), and diabetes mellitus (DM) (n = 7777) groups. Assessments during a median follow-up of 1681 days revealed that the cardiac mortality rates in the NGR, pre-DM, and DM groups were 4.9%, 5.4%, and 7.6%, respectively (*P* < 0.001) (Additional file 1: Fig. S1). The above research shows that AMI patients with glucose metabolism disorders can experience a high long-term risk of cardiac death, whereas there is no or very low risk of cardiac death among people without diabetes, indicating that patients with AMI undergoing PCI still have a large cardiovascular residual risk to further explore.

Malnutrition is common in people with coronary heart disease (CAD) with impaired glucose metabolism. It was demonstrated that obese yet malnourished [high BMI, low serum albumin (SA)] individuals not only often have diabetes but also carry the highest comorbidity burden, the most adverse cardiac remodeling, and the least favorable composite outcome [4]. Previous studies reported that more than 50% of CAD patients with diabetes had malnutrition, and the mortality rate of those patients with severe malnutrition was nearly doubled [5]. The prognostic nutritional index (PNI) is an important tool for objective malnutrition screening of the cardiovascular disease population and may become a new biomarker to predict long-term outcomes of CAD patients after PCI [6]. Another study showed that a low level of PNI was closely associated with increased acute kidney injury and mortality after PCI [7]. Additionally, it was demonstrated that a lower PNI is associated with all-cause mortality and heart failure hospitalizations in outpatients with preserved ejection fractions [8].

The level of impaired glucose metabolism and malnutrition could reflect the poor long-term prognosis of CAD patients undergoing PCI. Nevertheless, there are few studies investigating PNI in relation to the prognosis of AMI patients undergoing PCI with different glucose states. Our objective was to examine the relationship between PNI and long-term outcomes in patients with AMI undergoing PCI with different glucose metabolisms.

## Methods

### Study population

**Fig. 1:**
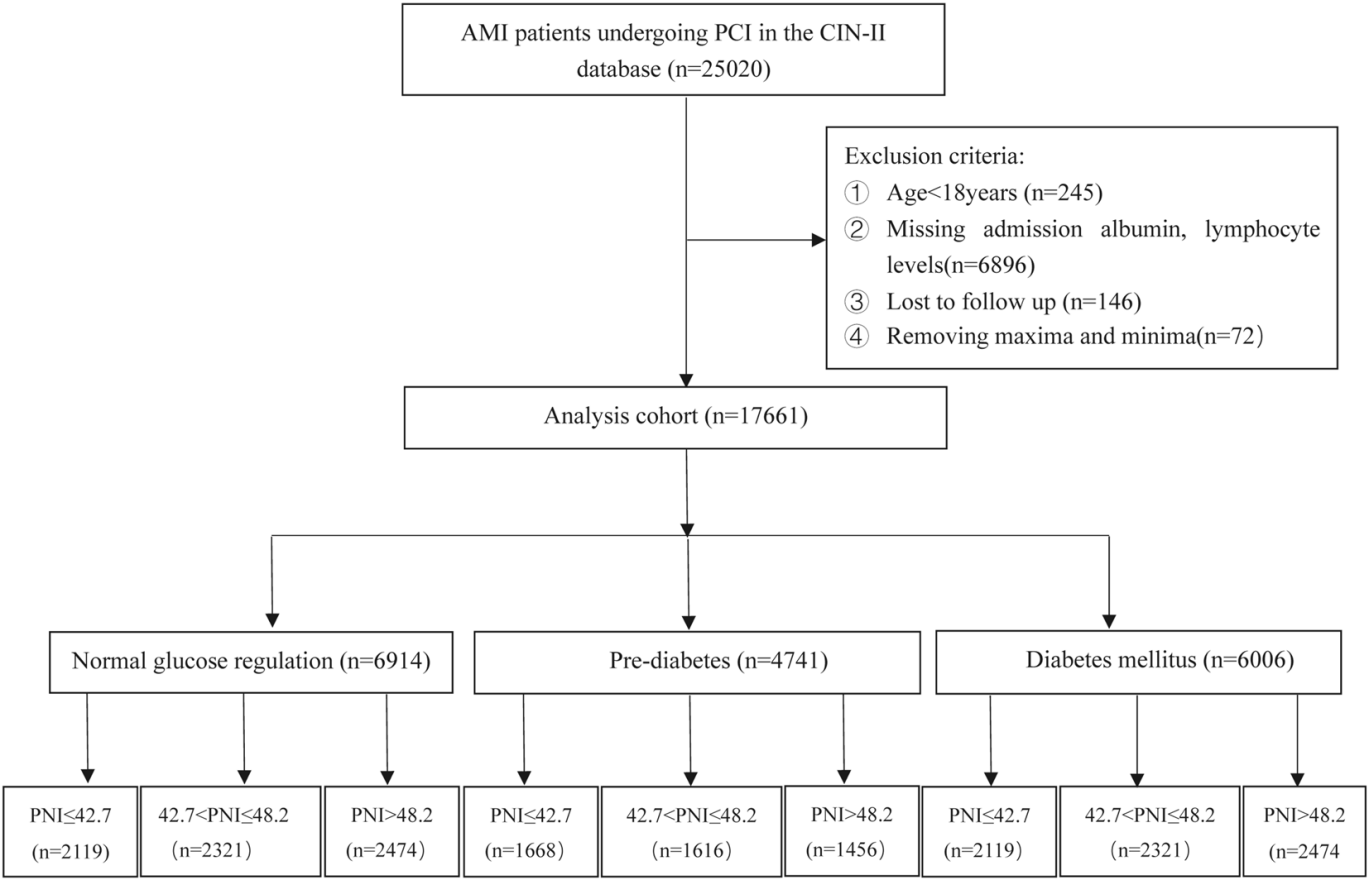
Flow diagram of patient selection. *PNI* prognostic nutritional index, *AMI* acute myocardial infarction, *PCI* percutaneous coronary intervention

The Cardiorenal Improvement II (CIN-II) study was a multi-center, retrospective, observational cohort study.  This database is based on the population of coronary angiography, and we enrolled 25,020 consecutive AMI patients who underwent PCI in Guangdong Provincial People’s Hospital, Guangdong, China, between January 2007 and December 2020 (ClinicalTrials.gov identifier NCT05050877). Study exclusion criteria included ① age < 18 years (n = 245), ② missing admission albumin or lymphocyte measurements (n = 6896), ③ lost to follow-up (n = 146), and ④ removal of maxima and minima (n = 72). Eventually, a total of 17,661 patients were included (Fig. 1). AMI concluded acute ST-elevation myocardial infarction and acute non–ST-elevation myocardial infarction, and the diagnosis, indication for PCI, and drug treatment were determined following standard clinical practice guidelines [9].

### Collection of clinical and demographic characteristics

From January 2007 to December 2020, data were extracted from the CIN-II study database. The baseline information included demographic characteristics, coexisting conditions, laboratory examinations, and medications at discharge. Patients were advised to fast for at least 12 h before fasting plasma glucose (FPG) and blood lipid profile measurements were collected, and other values were all collected at admission or before PCI. The laboratory data included plasma and biochemical parameters, such as levels of hemoglobin A1c (HbA1c), serum creatinine, triglycerides, low-density lipoprotein cholesterol, high-density lipoprotein cholesterol, serum albumin (ALB), fibrinogen, total lymphocyte count (LYM), absolute neutrophil count, platelet count, and hemoglobin. The ratio of neutrophils to lymphocytes was calculated by dividing the absolute neutrophil count by the LYM. Medical history was assessed using International Classification of Diseases, 10th revision, codes, and 54.6% of patients underwent trans-thoracic echocardiography before or after PCI. The left atrial diameter, left ventricular end-diastolic dimension, and left ventricular end-systolic dimension were obtained in the parasternal long-axis view.

### Clinical definition

DM patients were defined by ① a previous history of diabetes or use of antidiabetic agents and ② FPG ≥ 7.0 mmol/L or HbA1c ≥ 6.5% at admission. Pre-DM patients were defined by an FPG of 5.6 to < 7.0 mmol/L or an HbA1c concentration of 5.7% to < 6.5% at admission, while NGR patients were defined as those without DM or pre-DM [10]. Anemia was defined by a hematocrit concentration of ≤ 39% (male) or ≤ 36% (female). The PNI score was calculated using the formula: 10 × ALB (g/dL) + 0.005 × LYM (per mm^3^). According to the tertiles of PNI, the whole study group was divided into the following three subgroups: PNI-L, PNI-M, and PNI-H. The three groups of NGR, pre-DM, and DM adopted a unified classification standard. CKD was defined by an estimated glomerular filtration rate (eGFR) < 60 mL/min/1.73 m^2^ according to the Chronic Kidney Disease Epidemiology Collaboration creatinine equation [11]. Congestive heart failure (CHF) was defined by Killip class > 1 or New York Heart Association functional classification > 2. Definitions of other variables, such as valvular heart disease, atrial fibrillation (AF), hypertension (HT), and stroke, were made using ICD-10 codes.

### Study endpoints

All-cause mortality and cardiovascular mortality events were  obtained from the official department for disease control and prevention. Other non-mortality events were adjudicated by trained nurses and research assistants through outpatient interviews and telephones according to pre-specified definitions [12]. The primary endpoint was long-term all-cause mortality. The secondary endpoint was long-term cardiovascular mortality.

### Statistical analysis

Baseline characteristics are presented as mean ± standard deviation values for continuous variables and proportions for categorical variables. The differences in baseline characteristics between groups were compared using Student’s *t*-test for continuous variables and chi-squared tests for categorical variables. The differences in characteristics in a box plot were compared using the Kruskal–Wallis test. Time-to-event data among groups are presented graphically using Kaplan–Meier curves and compared by the log-rank test. Cox proportional hazards models were used to analyze all-cause mortality and calculate hazard ratios (HRs) and 95% confidence intervals (CIs). Baseline variables exhibiting a *P*-value<0.0001, or those with clinical significance were incorporated into Cox regression models. The final model with adjustment for major covariables, including age, sex, HT, CHF, CKD, AF, stroke, and anemia, was made by stepwise selection method based on *P* < 0.05; then, it was refined to select clinically meaningful covariates.Competing risk model analyses were used to investigate the prognosis impact of cardiovascular mortality. Interactions between treatment and subgroups were examined using a test for heterogeneity, using *P* <0.05 as signifiacant. Presented tests were 2-tailed for all, and *P* < 0.05 was considered statistically significant. All statistical analyses were performed using R (ver. 4.2.1; R Foundation for Statistical Computing, Vienna, Austria).

## Results

### Baseline characteristics of the study population

A total of 17,661 AMI patients who underwent PCI were included in the analysis. Table 1 presents baseline characteristics for patients according to their baseline PNI level and glucose statuses. Patients with lower PNI levels were older, more likely to be female, and more often had DM or pre-DM. Additionally, these patients tended to have greater incidence rates of HT, CHF, CKD, AF, stroke, and anemia than those with higher PNI levels. Moreover, the PNI-L group had lower LVEF and eGFR values but higher hs-cTnT, NT-proBNP, FPG, hs-CRP, and HbA1c levels than the PNI-H group. Patients with DM were oldest and had the highest incidence rates of HT, CHF, CKD, AF, stroke, and anemia compared to those with pre-DM or NGR statuses. In addition, values of LVEF, PNI, and eGFR were lowest and those of hs-cTnT, NT-proBNP, FPG, and hs-CRP were highest in patients with DM (Table 1).

**Table 1:** Baseline characteristics of the patients according to baseline Prognostic nutritional index and glucose metabolism statuses

	Baseline PNI category			*p* value	Glucose metabolism statuses			*p* value
	PNI-L (PNI ≤ 42.7)	PNI-M (> 42.7, ≤ 48.2)	PNI-H (> 48.2)		NGR	pre-DM	DM	
No. of subjects	5910	5895	5856		6914	4741	6006	
Age, years	65.7 ± 11.4	61.5 ± 11.6	57.0 ± 11.9	< 0.0001	60.8 ± 12.9	61.5 ± 11.7	62.0 ± 11.6	< 0.0001
Age > 65, years	3154 (53.4)	2189 (37.1)	1406 (24.0)	< 0.0001	2578 (37.3)	1817 (38.3)	2355 (39.2)	0.0793
Female sex	1226 (20.7)	1092 (18.5)	864 (14.8)	< 0.0001	1096 (15.9)	705 (14.9)	1381 (23.0)	< 0.0001
Current smoking, n (%)	1699 (44.7)	1582 (48.0)	1630 (50.0)	< 0.0001	1992 (49.0)	1485 (51.6)	1435 (42.0)	< 0.0001
Hypertension, n (%)	3025 (51.2)	2817 (47.8)	2577 (44.0)	< 0.0001	2880 (41.7)	2129 (44.9)	3412 (56.8)	< 0.0001
Glucose metabolism status				< 0.0001				< 0.0001
NGR, n (%)	2119 (35.9)	2321 (39.4)	2474 (42.3)		6914 (100.0)	NA	NA	
pre-DM, n (%)	1668 (28.2)	1616 (27.4)	1456 (24.9)		NA	4741 (100.0)	NA	
DM, n (%)	2123 (35.9)	1957 (33.2)	1925 (32.9)		NA	NA	6006 (100.0)	
Congestive heart failure, n (%)	2700 (45.7)	1979 (33.6)	1706 (29.1)	< 0.0001	2351 (34.0)	1623 (34.2)	2412 (40.2)	< 0.0001
Chronic kidney disease, n (%)	2118 (35.8)	1143 (19.4)	754 (12.9)	< 0.0001	1322 (19.1)	971 (20.5)	1723 (28.7)	< 0.0001
Atrial fibrillation, n (%)	297 (5.0)	207 (3.5)	136 (2.3)	< 0.0001	232 (3.4)	164 (3.5)	244 (4.1)	0.0779
Proir stroke, n (%)	469 (7.9)	316 (5.4)	241 (4.1)	< 0.0001	318 (4.6)	246 (5.2)	462 (7.7)	< 0.0001
Hyperlipemia, n (%)	3626 (61.4)	3620 (61.4)	4124 (70.4)	< 0.0001	4195 (60.7)	3136 (66.1)	4040 (67.3)	< 0.0001
Anemia, n (%)	3262 (55.2)	1775 (30.1)	820 (14.0)	< 0.0001	2158 (31.2)	1504 (31.7)	2197 (36.6)	< 0.0001
Proir PCI, n (%)	264 (4.5)	238 (4.0)	230 (3.9)	0.3006	235 (3.4)	183 (3.9)	314 (5.2)	< 0.0001
Proir MI, n (%)	88 (1.5)	84 (1.4)	86 (1.5)	0.9573	73 (1.1)	67 (1.4)	119 (2.0)	0.0001
Proir CABG, n (%)	10 (0.2)	7 (0.1)	1 (0.0)	0.0313	7 (0.1)	2 (0.0)	9 (0.1)	0.2212
Procedure_DES, n (%)	5612 (95.0)	5625 (95.4)	5579 (95.3)	0.4597	6506 (94.1)	4538 (95.7)	5774 (96.1)	< 0.0001
Procedure_BES, n (%)	164 (2.8)	134 (2.3)	107 (1.8)	0.0027	198 (2.9)	105 (2.2)	102 (1.7)	0.0001
Procedure_CABG, n (%)	2 (0.0)	3 (0.1)	1 (0.0)	0.6098	1 (0.0)	3 (0.1)	2 (0.0)	0.3726
Hemoglobin, g/L	125.1 ± 19.2	136.2 ± 17.1	143.9 ± 16.1	< 0.0001	136.7 ± 19.0	135.4 ± 17.4	132.9 ± 20.4	< 0.0001
eGFR, mL/min/1.73 m^2^	68.6 (26.0)	79.3 (22.7)	85.1 (21.2)	< 0.0001	80.2 (22.5)	77.6 (22.4)	74.7 (27.4)	< 0.0001
LDL-C, mmol/L	2.8 ± 1.0	3.1 ± 1.0	3.5 ± 1.2	< 0.0001	3.2 ± 1.1	3.1 ± 1.0	3.1 ± 1.1	< 0.0001
HDL-C, mmol/L	1.0 ± 0.3	1.0 ± 0.3	1.1 ± 0.3	< 0.0001	1.1 ± 0.3	1.0 ± 0.3	1.0 ± 0.3	< 0.0001
TG, mmol/L	1.4 ± 0.8	1.6 ± 1.1	2.0 ± 1.6	< 0.0001	1.5 ± 1.1	1.6 ± 0.9	1.9 ± 1.5	< 0.0001
hs-cTnT, ng/L	1068.0 [203.5, 3112.8]	438.2 [31.2, 1885.2]	202.6 [12.7, 1134.8]	< 0.0001	405.6 [13.3, 2238.5]	594.6 [61.2, 2327.2]	601.5 [74.7, 2023.0]	< 0.0001
NT-proBNP, ng/L	1985.0 [837.4, 4472.0]	839.9 [347.9, 2090.5]	544.1 [203.3, 1298.5]	< 0.0001	863.2 [318.0, 2313.0]	904.6 [366.6, 2113.2]	1167.0 [436.5, 3065.5]	< 0.0001
FPG, mmol/L	7.5 ± 3.8	6.9 ± 3.1	6.9 ± 3.3	0.0002	5.4 ± 1.3	5.7 ± 1.3	9.0 ± 4.1	< 0.0001
HbA1c, %	6.7 ± 1.8	6.6 ± 1.7	6.6 ± 1.7	0.0308	5.3 ± 0.4	6.0 ± 0.2	8.0 ± 1.9	< 0.0001
hs-CRP, mg/L	36.0 ± 48.8	17.4 ± 31.8	11.8 ± 23.8	< 0.0001	18.4 ± 35.2	20.9 ± 35.6	24.0 ± 39.8	< 0.0001
FIB, g/L	5.1 ± 1.8	4.2 ± 1.5	3.8 ± 1.3	< 0.0001	4.1 ± 1.6	4.6 ± 1.7	4.6 ± 1.7	< 0.0001
ALB, g/L	32.1 ± 3.5	37.0 ± 2.8	41.0 ± 3.7	< 0.0001	37.4 ± 5.0	36.2 ± 4.7	36.2 ± 5.0	< 0.0001
PNI	38.6 ± 3.3	45.4 ± 1.6	52.7 ± 4.0	< 0.0001	46.0 ± 6.6	45.3 ± 6.4	45.2 ± 6.7	< 0.0001
PLT, 109/L	230.6 ± 81.7	239.1 ± 75.7	251.2 ± 69.2	< 0.0001	237.9 ± 76.0	242.3 ± 74.7	241.4 ± 77.6	0.0037
NEU, 10^9^/L	7.7 ± 3.8	7.6 ± 3.6	7.9 ± 3.7	0.0002	8.0 ± 3.8	7.3 ± 3.5	7.8 ± 3.8	< 0.0001
LYM, 10^9^/L	1.3 ± 0.5	1.7 ± 0.5	2.3 ± 0.8	< 0.0001	1.7 ± 0.8	1.8 ± 0.8	1.8 ± 0.8	< 0.0001
NLR	7.4 (7.1)	5.3 (4.0)	3.9 (2.8)	< 0.0001				
Left atrial, mm	36.5 ± 5.2	35.6 ± 4.6	35.3 ± 4.5	< 0.0001	35.3 ± 4.9	35.6 ± 4.7	36.8 ± 4.8	< 0.0001
LVESD, mm	35.3 ± 7.8	33.2 ± 6.7	32.4 ± 6.2	< 0.0001	33.2 ± 6.8	33.7 ± 7.1	34.4 ± 7.3	< 0.0001
LVEDD, mm	50.1 ± 6.3	48.9 ± 5.6	48.5 ± 5.5	< 0.0001	48.9 ± 5.7	49.2 ± 5.9	49.5 ± 6.0	0.0003
LVEF, %	51.7 ± 11.6	56.0 ± 10.5	57.9 ± 9.6	< 0.0001	56.1 ± 10.7	55.1 ± 10.5	54.0 ± 11.4	< 0.0001
Beta-blockers, n (%)	4400 (80.1)	4621 (83.9)	4575 (84.2)	< 0.0001	5076 (80.8)	3798 (83.1)	4723 (84.6)	< 0.0001
Statin, n (%)	5297 (96.4)	5365 (97.4)	5318 (97.9)	< 0.0001	6127 (97.5)	4462 (97.7)	5392 (96.5)	0.0005
CCB, n (%)	967 (17.6)	1103 (20.0)	1132 (20.8)	0.0001	925 (14.7)	824 (18.0)	1453 (26.0)	< 0.0001
ACEI or ARB, n (%)	3837 (69.8)	4039 (73.3)	3861 (71.1)	0.0002	4268 (67.9)	3345 (73.2)	4125 (73.9)	< 0.0001
Dual-antiplatelet therapy, n (%)	5156 (93.8)	5277 (95.8)	5236 (96.4)	< 0.0001	5981 (95.2)	4419 (96.7)	5270 (94.4)	< 0.0001

*PNI* prognostic nutritional index, *DM* diabetes mellitus, *pre-DM* pre-diabetes mellitus, *NGR* normal glucose regulation, *prior PCI* prior percutaneous coronary intervention, *prior MI* prior myocardial infarction, *prior CABG* prior coronary artery bypass graft, *HbA1C* hemoglobin A1c, *FPG* fasting plasma glucose, *eGFR* estimated glomerular filtration rate, *LDL-C* low density lipoprotein cholesterol, *HDL-C* high density lipoprotein cholesterol, *ALB* serum albumin, *FIB* fibrinogen, *LYM* total lymphocyte count, *NEU* absolute neutrophil count, *PLT* platelet count, *NLR* neutrophil-to-lymphocyte ratio, *LVEF* left ventricular ejection fraction, *LVEDD* left ventricular end-systolic dimension, *LVESD* left ventricular end-systolic dimension, *ACEI* angiotensin converting enzyme inhibitor, *ARB* angiotensin receptor blocker, *CCB* calcium block

### Ability of PNI to predict all-cause mortality and cardiovascular mortality

**Fig. 2:**
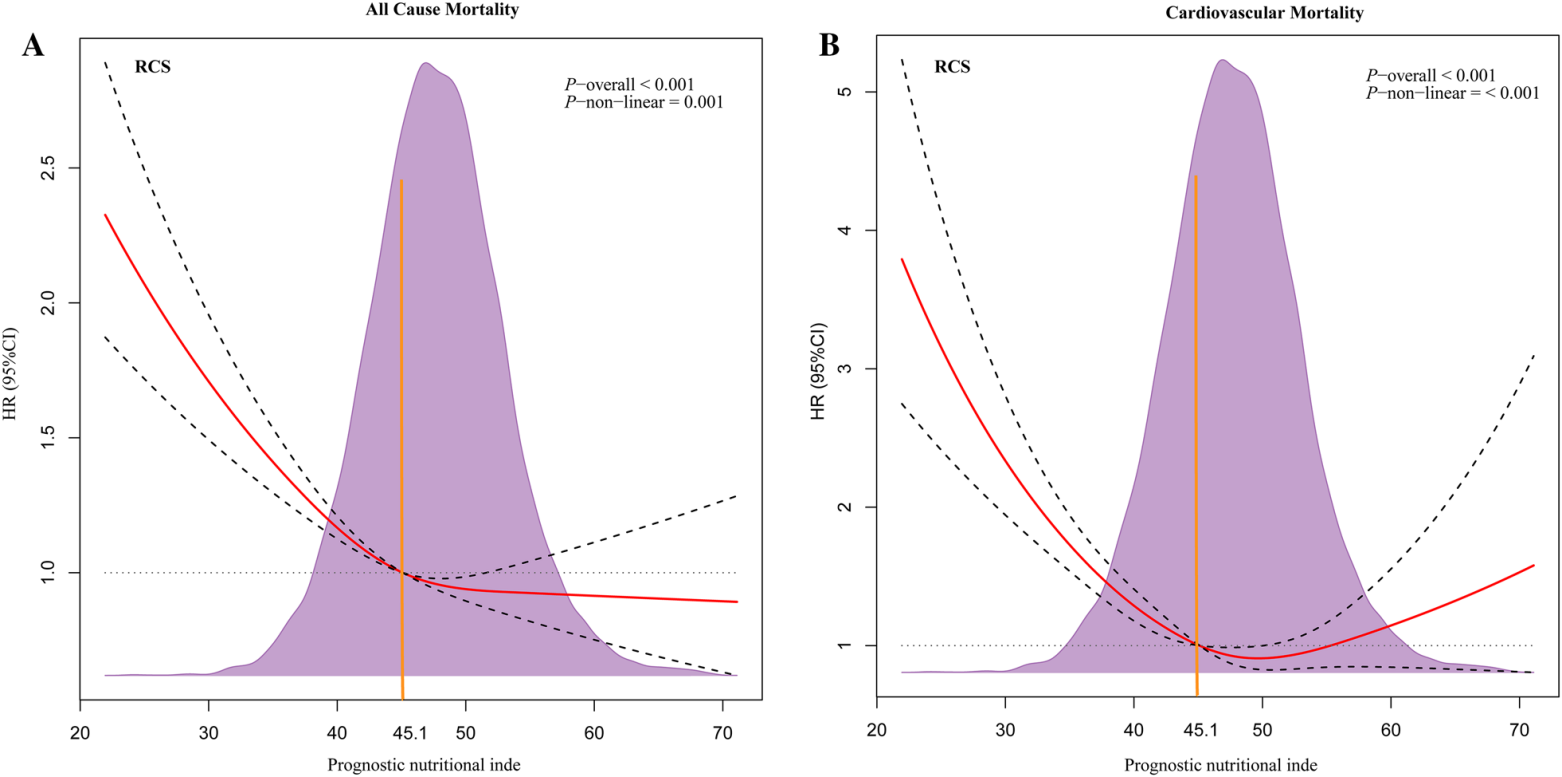
Restricted cubic spline regression analysis of PNI index within relation to all-cause mortality **A** and cardiovascular mortality **B**. Values were adjusted for age, gender, sex, HT, CHF, CKD, AF, stroke, and anemia. *RCS*, restricted cubic spline; *HR*, hazard ratio; *CI*, confidence interval

**Fig. 3:**
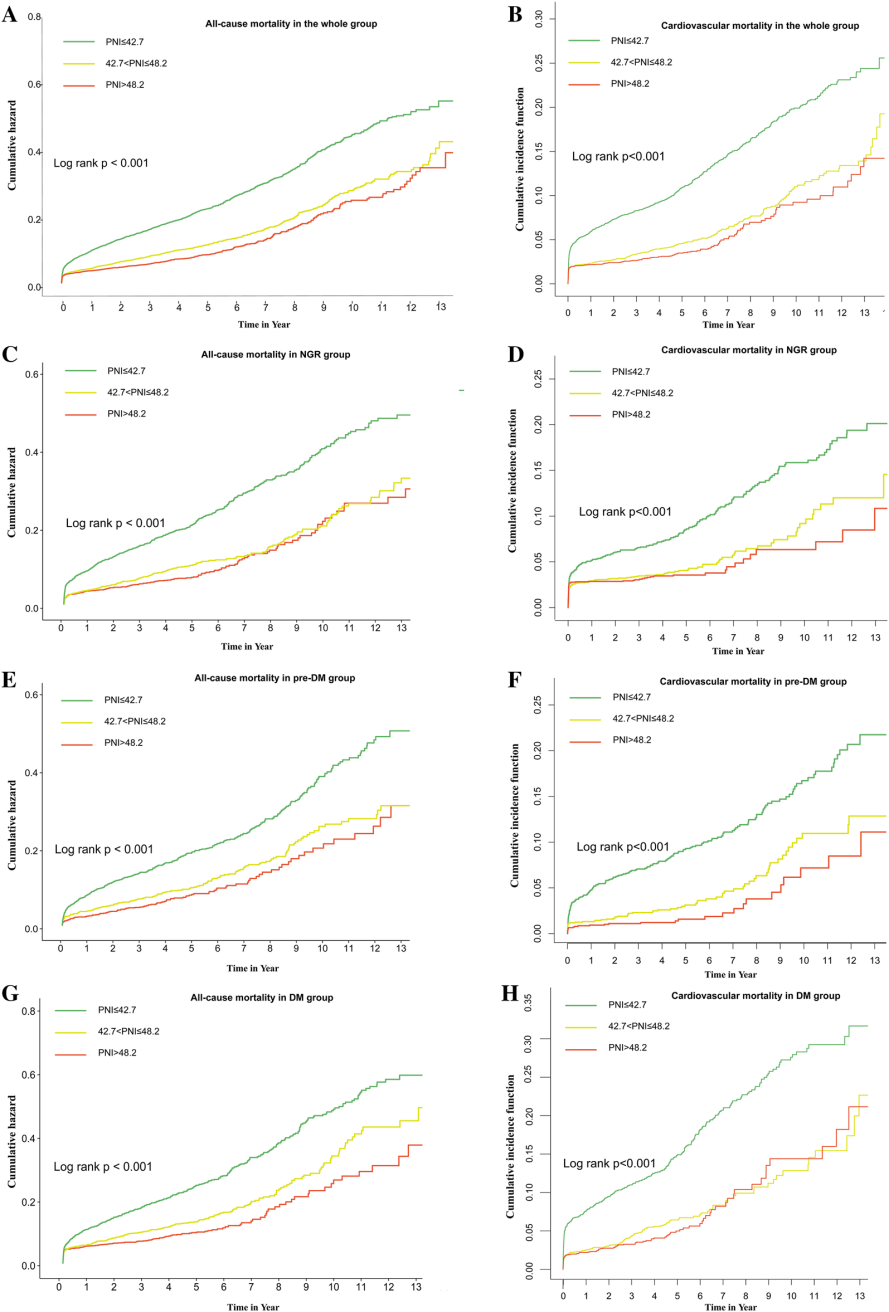
Cumulative incidences of all-cause mortality (Kaplan-–Meier curve) and cardiac mortality (cumulative incidence function curve) risks for patients with different glucose statuses. Cumulative hazard of all‑cause mortality **A** and cardiovascular mortality **B** across the whole group with different PNI index; Cumulative hazard of all‑cause mortality **C** and cardiovascular mortality **D** across the NGR group with different PNI index; Cumulative hazard of all‑cause mortality **E** and cardiovascular mortality **F** across the pre-DM group with different PNI index; Cumulative hazard of all‑cause mortality **G** and cardiovascular mortality **H** across the DM group with different PNI index. *PNI*, prognostic nutritional index; *DM*, diabetes mellitus; *pre-DM*, pre-diabetes mellitus; *NGR*, normal glucose regulation

Restricted cubic spline models showed that a J-shaped association existed between PNI and long-term all-cause or cardiovascular mortality, and the relationship of both was non-linear (non-linear *P* = 0.001 or *P* < 0.001) (Fig. 2). A PNI index of 45.1 was selected as the reference level represented by the vertical dotted lines. Lower levels of PNI were associated with increased risks of all-cause and cardiovascular mortality. All-cause mortality occurred in 2613 (14.8%) patients and cardiovascular mortality occurred in 1222 (6.9%) patients during a median of 4.1 years of follow-up. The multivariable analysis showed that the PNI-L subgroup among NGR, pre-DM, and DM patients had a similar heightened risk of al-cause mortality compared to the PNI-H subgroup (adjusted HR, 1.35; 95% CI 1.2–1.51; *P* < 0.001) (Table 2). PNI-L increased the risk of cardiovascular mortality in DM patients (HR, 1.316; 95% CI 1.009–1.717; *P* = 0.043) and pre-DM patients HR, 1.765; 95% CI 1.147–2.716; *P* = 0.01), while no significant difference was found in the NGR group (*P* > 0.05) (Table 3). Moreover, there was no significant difference between the PNI-M and PNI-H subgroups among NGR, pre-DM, and DM patients. The Kaplan–Meier survival curve showed the same trends for the relationship between PNI and all-cause mortality of different glucose statuses. The cumulative hazard in the PNI-L group was significantly higher than those of other groups (log-rank *P* < 0.001) (Fig. 3A, C, E, and G). Similarly, the competing risk analysis showed that PNI-L statuses was associated with cardiac death after adjusting for a competing event (non-cardiac death) (Fine–Gray test *P* < 0.001) (Fig. 3B, D, F, and H).

**Table 2:** The HR (95% CI) of all-cause mortality according to PNI and different glucose metabolism statuses from the three models

Glucose regulation state	PNI	Events, n (%)	Model 1		Model 2		Model 3	
			HR (95% CI)	*P* value	HR (95% CI)	*P* value	HR (95% CI)	*P* value
Total	High	486/5855 (8.3)	Ref.		Ref.		Ref.	
	Median	714/5894 (12.1)	1.32 (1.17, 1.48)	< 0.001	1.11 (0.99, 1.25)	0.07	1.05 (0.94, 1.18)	0.39
	Low	1411/5910 (23.9)	2.33 (2.1, 2.59)	< 0.001	1.65 (1.48, 1.84)	< 0.001	1.35 (1.2, 1.51)	< 0.001
NGR	High	183/2474 (7.4)	Ref.		Ref.		Ref.	
	Median	262/2321 (11.3)	1.37 (1.13, 1.65)	0.001	1.15 (0.95, 1.4)	0.14	1.14 (0.94, 1.38)	0.17
	Low	449/2119 (21.2)	2.19 (1.84, 2.61)	< 0.001	1.55 (1.29, 1.85)	< 0.001	1.38 (1.14, 1.66)	< 0.001
pre-DM	High	115/1456 (7.9)	Ref.		Ref.		Ref.	
	Median	194/1616 (12.0)	1.31 (1.04, 1.64)	0.02	1.07 (0.85, 1.35)	0.57	1.01 (0.8, 1.28)	0.94
	Low	379/1669 (22.7)	2.22 (1.8, 2.74)	< 0.001	1.49 (1.2, 1.87)	< 0.001	1.29 (1.02, 1.62)	0.03
DM	High	188/1925 (9.8)	Ref.		Ref.		Ref.	
	Median	258/1957 (13.2)	1.25 (1.03, 1.51)	0.02	1.08 (0.89, 1.31)	0.42	0.98 (0.81, 1.19)	0.85
	Low	583/2126 (27.5)	2.47 (2.09, 2.92)	< 0.001	1.85 (1.56, 2.19)	< 0.001	1.36 (1.13, 1.63)	< 0.001

*PNI* prognostic nutritional index, *DM* diabetes mellitus, *pre-DM* pre-diabetes mellitus, *NGR* normal glucose regulation, *Ref.* reference, *HR* hazard ratio, *CI* confidence interval

High: PNI > 48.2; median: 42.7 < PNI ≤ 48.2; low: PNI ≤ 42.7

Model 1: unadjusted

Model 2: adjusted for model 1 + age + gender

Model 3: adjusted for model 2 + HT + CHF + CKD + AF + stroke + anemia

**Table 3:** The HR (95% CI) of cardiovascular mortality according to PNI and different glucose metabolism states from the three models

Glucose regulation state	PNI	Events, n (%)	Model 1		Model 2		Model 3	
			HR (95% CI)	*P* value	HR (95% CI)	*P* value	HR (95% CI)	*P* value
Total	High	207/5856 (3.5)	Ref.		Ref.		Ref.	
	Median	297/5895 (5.0)	1.209 (1.009, 1.449)	0.04	1.027 (0.857, 1.231)	0.774	0.97 (0.808, 1.166)	0.748
	Low	716/5910 (12.1)	2.24 (1.897, 2.644)	< 0.001	1.619 (1.364, 1.921)	< 0.001	1.296 (1.085, 1.549)	0.004
NGR	High	88/2474 (3.6)	Ref.		Ref.		Ref.	
	Median	109/2321 (4.7)	1.133 (0.848, 1.514)	0.399	0.95 (0.71, 1.27)	0.727	0.949 (0.708, 1.27)	0.724
	Low	213/2119 (10.1)	1.848 (1.418, 2.408)	< 0.001	1.318 (0.999, 1.74)	0.051	1.209 (0.906, 1.612)	0.197
pre-DM	High	28/1456 (1.9)	Ref.		Ref.		Ref.	
	Median	67/1616 (4.1)	1.772 (1.141, 2.752)	0.011	1.39 (0.896, 2.157)	0.142	1.236 (0.79, 1.932)	0.354
	Low	176/1669 (10.6)	3.671 (2.44, 5.524)	< 0.001	2.185 (1.43, 3.339)	< 0.001	1.765 (1.147, 2.716)	0.01
DM	High	91/1925 (4.7)	Ref.		Ref.		Ref.	
	Median	121/1957 (6.2)	1.772 (1.141, 2.752)	0.011	1.001 (0.759, 1.32)	0.996	0.932 (0.704, 1.235)	0.625
	Low	327/2124 (15.4)	3.671 (2.44, 5.524)	< 0.001	1.775 (1.382, 2.28)	< 0.001	1.316 (1.009, 1.717)	0.043

*PNI* prognostic nutritional index, *DM* diabetes mellitus, *pre-DM* pre-diabetes mellitus, *NGR* normal glucose regulation, *Ref.* reference, *HR* hazard ratio, *CI* confidence interval

High: PNI > 48.2; median: 42.7 < PNI ≤ 48.2; low: PNI ≤ 42.7

Model 1: unadjusted

Model 2: adjusted for model 1 + age + gender

Model 3: adjusted for model 2 + HT + CHF + CKD + AF + stroke + anemia

### Subgroup analysis

**Fig. 4:**
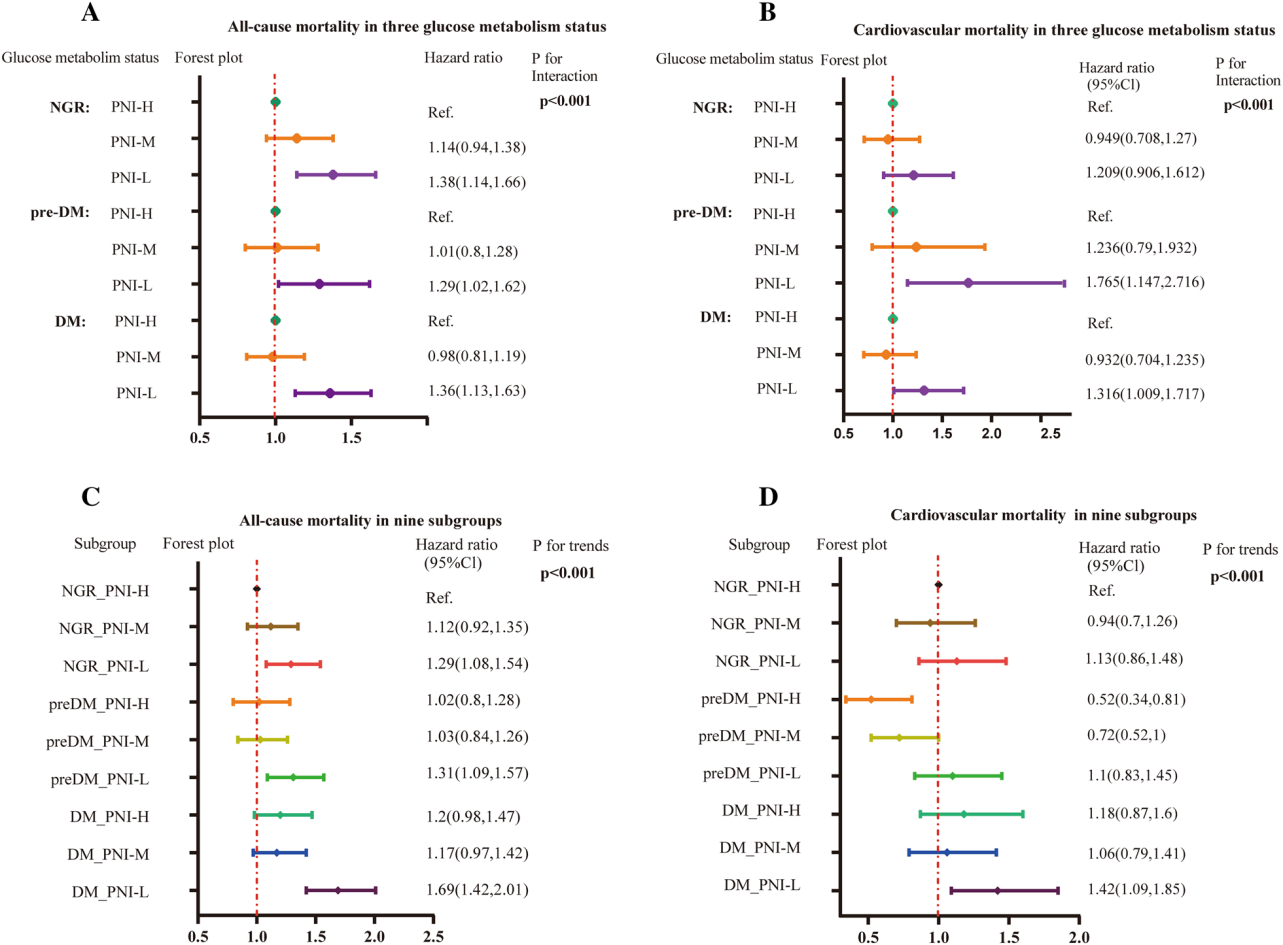
Hazard ratios for long-term all-cause **A** and cardiovascular mortality **B** in different glucose metabolism statuses. Hazard ratios for long-term all-cause **C** and cardiovascular mortality **D** in different subgroups. *PNI*, prognostic nutritional index; *DM*, diabetes mellitus; *pre-DM*, pre-diabetes mellitus; *NGR*, normal glucose regulation; *Ref.*, Reference; *CI*, confidence interval; *PNI-H*: PNI>48.2; *PNI-M*: 42.7

The results of subgroup analysis were in line with those of the overall analysis, and formal testing for interactions showed that the relative risks of 4.1-year all-cause mortality in a comparison between the high PNI and low PNI groups were consistent across almost all subgroups among patients with DM, pre-DM, or NGR. The relationship between PNI and a risk of all-cause mortality was modified by glucose metabolism statuses (*P* for interaction < 0.001) (Fig. 4A). Different glucose metabolism also had interaction with PNI with respect to cardiovascular mortality (*P* for interaction < 0.001) (Fig. 4B). Therefore, we further assessed for mortality risk in the nine subgroups due to the interaction between PNI and glucose metabolism statuses, which was independently associated with the risk of all-cause and cardiovascular mortality (Fig. 4C, D). Especially, patients with PNI-L and DM were 1.69 times more at risk for all-cause mortality than patients with PNI-H or NGR statuses (adjusted HR, 1.69; 95% CI 1.42–2.01).

## Discussion

To our knowledge, this is the first large real-world cohort study to explore the relationship between nutritional statuses and glucose statuses among patients with AMI undergoing PCI in China. The main finding of our study is prevalent among patients with glucose metabolism disorder, even in DM patients, and is strongly associated with increased mortality.

As the most serious state of blood glucose metabolism disorder, DM is closely related to the progression of coronary heart disease and a poor prognosis [13]. The progression of diabetes to coronary artery disease is mainly related to hyperglycemia, dyslipidemia, changes in the secretion of hormones other than insulin, and a pro-inflammatory statuses, and oxidative stress and inflammation interact to promote an abnormal glucose metabolism statuses and accelerate atherosclerosis [14]. Cardiovascular disease is the most common cause of death for DM patients, and AMI patients are more likely to be associated with blood glucose metabolism disorder. Studies show that the annual incidence of insulin resistance in patients with an AMI history is twice that in patients without an AMI history [15]. Several factors may explain the worse long-term outcome of AMI patients with DM, including a higher coronary atherosclerotic burden, more vulnerable plaques, and enhanced platelet reactivity [16, 17]. Although pre-DM patients tend to have a mild glucose metabolism disorder, more and more studies have confirmed that it is significantly related to cardiovascular events. Only 5–10% of pre-DM patients develop DM every year, while the same proportion will be converted into an NGR statuses. The division of these three blood glucose states is mainly based on the level of HbA1c, and it was found that every 1% increase in HbA1c was associated with a greater risk of cardiovascular events, with an HR of 1.07 [18]. The increase in the HbAlc level can increase the viscosity of red blood cells, heighten the occurrence of thrombotic events, and change the affinity of red blood cells for oxygen, leading to hypoxia of myocardial cells, aggravating the pathological changes of cardiac microvessels, and causing an abnormal myocardial metabolism. Therefore, grouping research based on blood glucose statuses is a preferable approach for evaluating the disease progression and prognosis of AMI patients who underwent PCI.

Although AMI patients with PCI have undergone timely blood supply reconstruction, they still experience high long-term all-cause and cardiovascular mortality rates, suggesting that there are many residual cardiovascular risks still to be identified. Therefore, in addition to focusing on traditional cardiovascular risk factors, the identification and intervention of non-traditional factors will help to play an important role in understanding the disease progression and prognosis of the AMI with PCI history population. Recent studies have shown that non-traditional cardiovascular risk factors represented by malnutrition are closely related to a poor prognosis of AMI patients. At present, the scoring indicators of malnutrition mainly include PNI; the Geriatric Nutritional Risk Index (GNRI); and triglyceride, total cholesterol, and body weight index scores [19]. PNI can provide nutritional, inflammatory, and immunological information, is more and more widely used to evaluate the prognosis of the AMI population, can be used as an important indicator of the prognosis of patients with congestive heart failure [20], and has a high predictive value for long-term cardiovascular and cerebrovascular events in the CAD intervention population [6]. To our knowledge, there are no specific large-scale studies considering the prognostic value of PNI in different glucose metabolism statuses groups, and glucose metabolism disorder leads to high cardiovascular risks and should be paid more attention. In our cohort, the all-cause mortality of NGR patients was 12.9%, that of pre-DM patients was 14.5%, and that of DM patients was as high as 17.1%, with all cases significantly associated with malnutrition. In contrast, DM patients with PNI-L had the highest risk of all-cause mortality, which could be up to 1.36 times higher than that of DM patients with PNI-H and 1.69 times higher than that of NGR patients with PNI-H. Due to disorders of glucose and lipid metabolism, diet control, and the influence of hypoglycemic drugs, DM patients are more likely to suffer from malnutrition. After the occurrence of AMI, gastrointestinal absorption dysfunction leads to a nutrient intake decline, reducing the stability of the internal environment and the reserve function of organs, leaving the nutritional needs of the body unsatisfied; moreover, patients with nutritional risks also have poor drug resistance and experience obstacles in food intake and nutrient absorption, which can worsen the nutritional statuses of AMI patients. Evidence shows that the blood glucose level has a significant impact on the nutritional statuses of patients with CAD and is more likely to lead to the aggravation of coronary artery lesions and cardiovascular events [21]. Additionally, malnutrition associated with low serum albumin at admission is independently associated with a greater risk for bleeding events in patients with AMI undergoing PCI [22]. DM can aggravate the inflammatory reaction process of coronary atherosclerosis, while long-term chronic inflammation aggravates the occurrence of malnutrition; subsequently, malnutrition and inflammation form a vicious circle, increasing the risk of long-term cardiovascular death in patients with AMI. Therefore, AMI combined with malnutrition can further aggravate myocardial injury and inflammatory reactions, which is consistent with the findings that troponin, BNP, and CRP significantly increased and LVEF significantly decreased in the PNI-L subgroup at the baseline during our study. Additionally, DM patients with malnutrition could also show an association with carcinogenesis, cancer progression, and death [23].

In the AMI population without a DM history, more than 50% of patients were diagnosed with pre-DM [24]. A disorder of glucose metabolism exists in the pre-DM state, but clinical interventions for this population are fewer in number and more easily ignored. Previous studies have reported that AMI patients with pre-DM are older, had higher BMIs and worse cardiac function, and were more likely to be associated with multiple cardiovascular risk factors, which became an independent predictor of cardiovascular events in AMI [25]. Additionally, pre-DM has a similar impact as DM on the long-term prognosis of major adverse cardiovascular events in patients with myocardial infarction with non-obstructive coronary arteries [26]. In this study, we found that the long-term all-cause mortality risk of the PNI-L subgroup was significantly different in the pre-DM population. Interestingly, there was a greater risk of cardiovascular mortality in the PNI-L subgroup than the total DM population. In addition, it was found that LYMs from the pre-DM group were lower than those from the DM and NGR groups. Coronary atherosclerosis rupture, acute thrombosis, and systemic inflammatory reactions are important mechanisms of AMI. MIRI after revascularization can further aggravate the damage caused by cardiac inflammatory reactions [1]. Lymphocytes, as a core component of the adaptive immune response, participate in inflammatory reactions in the process of atherosclerosis and play anti-inflammatory and anti-atherosclerosis roles [27]. The study confirmed that the level of T_regs_ in patients with AMI decreased, which is closely related to cardiovascular events [28]. In the acute phase of coronary events, the increase in corticosteroid hormones causes a stress reaction, leading to a large trend of lymphocyte apoptosis, weakening the anti-inflammatory effect, which can lead to an increased risk of cardiovascular events [29]. Leukocytes are an independent predictor of inflammation and the immune response. Neutrophils can cause vascular endothelial dysfunction and vascular wall degradation by secreting inflammatory cytokines. The ratio of neutrophils to lymphocytes has gradually been considered as a reliable indicator of systemic inflammation and the immune response [30]. Our study shows that the highest ratio of neutrophils to lymphocytes in the PNI-L subgroup indicates the most serious inflammatory state. Moreover, DM patients show inhibition of the immune response, while pre-DM patients start to show upregulation of inflammatory markers and immune activation [31]. Therefore, PNI has a significant value in predicting the risk of cardiac death in the pre-DM population.

Our study had several limitations. First, despite including a large sample size, this was a single-center study; thus, generalization of the findings should be done cautiously. Second, laboratory parameters were are not all checked at admission, which might cause potential bias due to measurement errors in different time periods. Third, DM was defined according to the ICD-10 code recorded at admission. Although there are some undetected DM patients that might have been enrolled in our study, this real-world study can also reflect the current situation of DM diagnosis. In addition, prospective cohort studies are required to confirm our findings.

## Conclusion

At present, malnutrition is common in patients with AMI undergoing PCI in different glucose states, especially DM patients, which can significantly increase their long-term mortality. Therefore, it is particularly important to conduct a hierarchical assessment of nutritional statuses, and early intervention for malnutrition will help improve the prognosis of patients with different glucose metabolism statuses. Our study firstly indicated that a lower PNI is a significant and independent risk factor for all-cause mortality in AMI patients undergoing PCI with different glucose metabolism statuses. Moreover, this risk was further increased in the DM group compared to the NGR and pre-DM groups. Pre-DM patients also had a worse risk of cardiovascular mortality. Further study is required to explore whether effective PNI increases can improve the prognosis in AMI patients undergoing PCI.

### Supplementary Information

**Additional file 1: Figure S1.** Cumulative incidences of cardiac mortality (cumulative incidence function curve) risks for AMI patients with different glucose statuses.

## Data Availability

The datasets generated and analyzed during the current study are not publicly available due to the institution policy but are available from the corresponding author on reasonable request.

## Abbreviations

PNI: Prognostic nutritional index
DM: Diabetes mellitus
pre-DM: Pre-diabetes mellitus
NGR: Normal glucose regulation
Prior PCI: Prior percutaneous coronary intervention
Prior MI: Prior myocardial infarction
Prior CABG: Prior coronary artery bypass graft
HbA1C: Hemoglobin A1c
FPG: Fasting plasma glucose
eGFR: Estimated glomerular filtration rate
LDL-C: Low density lipoprotein cholesterol
HDL-C: High density lipoprotein cholesterol
ALB: Serum albumin
FIB: Fibrinogen
LYM: Total lymphocyte count
NEU: Absolute neutrophil count
PLT: Platelet count
NLR: Neutrophil-to-lymphocyte ratio
LVEF: Left ventricular ejection fraction
LVEDD: Left ventricular end-systolic dimension
LVESD: Left ventricular end-systolic dimension
ACEI: Angiotensin converting enzyme inhibitor
ARB: Angiotensin receptor blocker
CCB: Calcium block
Ref.: Reference
HR: Hazard ratio
CI: Confidence interval
RCS: Restricted cubic spline
