# Supplementary figures and images for: Prognostic value of the prognostic nutritional index for patients with acute myocardial infarction undergoing percutaneous coronary intervention with variable glucose metabolism statuses: a retrospective cohort study
Source: Diabetol Metab Syndr. 2023 Oct 24;15:207. doi: 10.1186/s13098-023-01160-4 (PMC10594916; doi:10.1186/s13098-023-01160-4)

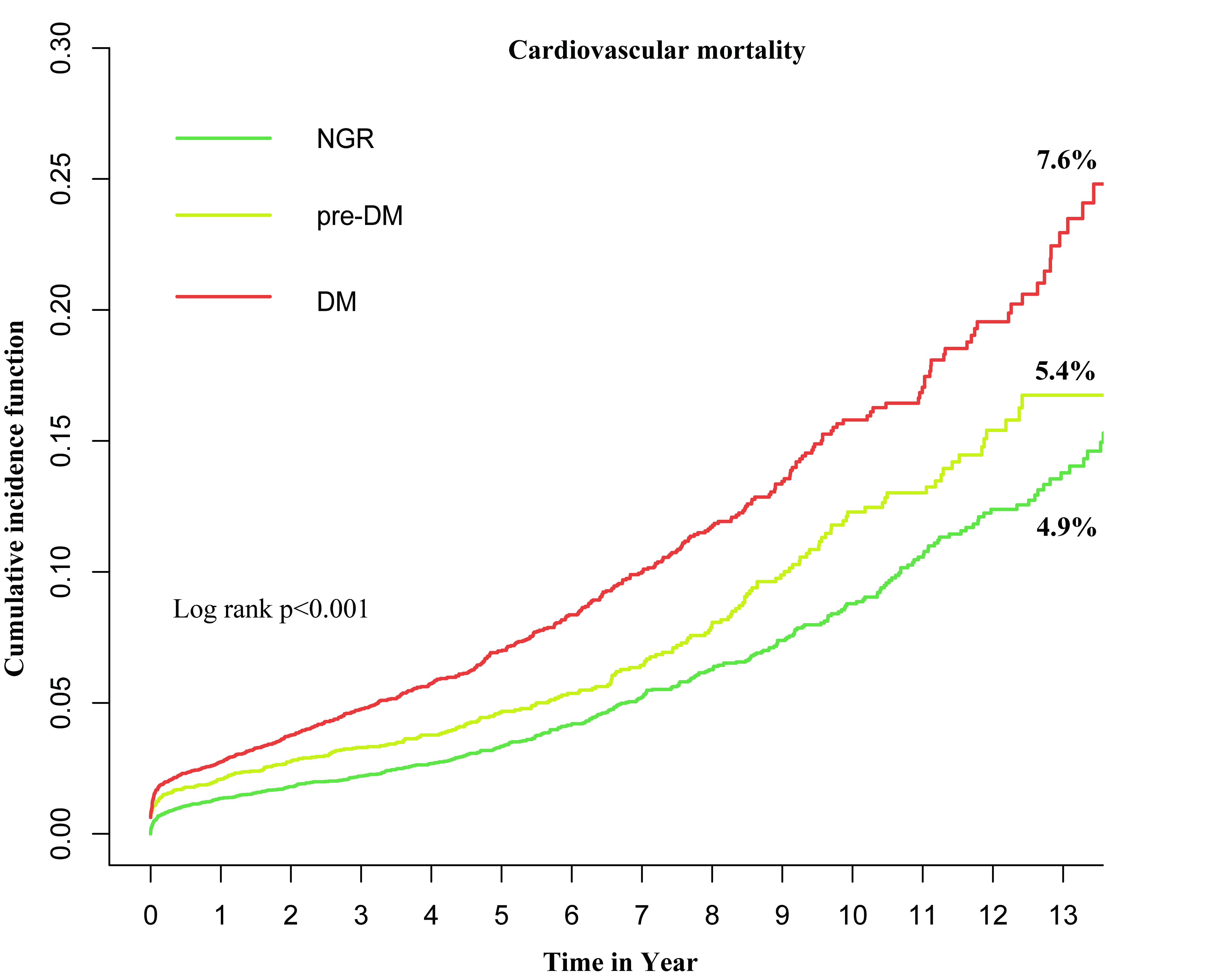

Supplement: Supplementary file 1 — Additional file 1: Figure S1. Cumulative incidences of cardiac mortality (cumulative incidence function curve) risks for AMI patients with different glucose statuses. [file 13098_2023_1160_MOESM1_ESM.tif]
